# Follistatin-like 1 protects mesenchymal stem cells from hypoxic damage and enhances their therapeutic efficacy in a mouse myocardial infarction model

**DOI:** 10.1186/s13287-018-1111-y

**Published:** 2019-01-11

**Authors:** Han Shen, Guanghao Cui, Yanqiong Li, Wenxue Ye, Yimin Sun, Zihan Zhang, Jingjing Li, Guiying Xu, Xiansheng Zeng, Yanxia Zhang, Wencheng Zhang, Zan Huang, Weiqian Chen, Zhenya Shen

**Affiliations:** 1 Institute for Cardiovascular Science and Department of Cardiovascular Surgery of the First Affiliated Hospital, Soochow University, Suzhou, 215006 China; 2 Department of Cardiovascular Surgery of the First Affiliated Hospital and Institute for Cardiovascular Science, Soochow University, Suzhou, 215006 China; 3 Department of Cardiology of the First Affiliated Hospital, Soochow University, Suzhou, 215006 China; 4 The Key Laboratory of Cardiovascular Remodeling and Function Research, Chinese Ministry of Education and Chinese Ministry of Health, Qilu Hospital of Shandong University, Jinan, China; 5 Jiangsu Province Key Laboratory of Gastrointestinal Nutrition and Animal Health, College of Animal Science and Technology, Nanjing Agriculture University, Nanjing, 210000 China

**Keywords:** Mesenchymal stem cells, Myocardial infarction, Transplantation, Follistatin-like 1, Survival

## Abstract

**Background:**

Cell therapy remains the most promising approach against ischemic heart injury. However, poor survival of engrafted cells in ischemic sites diminishes its therapeutic efficacy. Follistatin-like 1 (Fstl1) is documented as a novel pro-survival cardiokine for cardiomyocytes, and it is protective during ischemic heart injury. In the present study, we characterize the potential of Fstl1 as an effective strategy to enhance hypoxia resistance of donor cells and optimize stem cell-based therapy.

**Methods:**

Murine bone marrow-derived mesenchymal stem cells (MSCs) were expanded in monolayer culture and characterized by flow cytometry. MSCs were subjected to hypoxia to mimic cardiac ischemic environment. Expression of Fstl1 was monitored 0, 24, and 48 h following hypoxia. Constitutive expression of Fstl1 in MSCs was achieved by lentivirus-mediated Fstl1 overexpression. Genetically modified MSCs were further collected for cell death and proliferation assay following 48 h of hypoxic treatment. Acute myocardial infarction (MI) model was created by ligating the left anterior descending coronary artery, while control MSCs (MSCs-mCherry) or Fstl1-overexpressing MSCs (MSCs-Fstl1) were injected into the peri-infarct zone simultaneously. Subsequently, retention of the donor cells was evaluated on post-therapy 1, 3, & 7 days. Finally, myocardial function, infarct size, inflammation, and neovascularization of the infarcted hearts were calculated thereafter.

**Results:**

Expression of Fstl1 in hypoxic MSCs declines dramatically in a time-dependent manner. In vitro study further demonstrated that Fstl1 promotes survival and proliferation of hypoxic MSCs. Moreover, Fstl1 significantly prolongs MSC survival/retention after implantation. Finally, transplantation with Fstl1-overexpressing MSCs significantly improves post-MI cardiac function by limiting scar formation, reducing inflammatory response, and enhancing neovascularization.

**Conclusions:**

Our results suggest Fstl1 is an intrinsic cardiokine promoting survival and proliferation of MSCs, thereby optimizing their engraftment and therapeutic efficacy during cell therapy.

**Electronic supplementary material:**

The online version of this article (10.1186/s13287-018-1111-y) contains supplementary material, which is available to authorized users.

## Background

Myocardial infarction (MI) is the leading cause of morbidity and mortality worldwide. In terms of current therapeutic options for MI, stem cell-based therapies hold great promise for heart regeneration [1]. Bone marrow-derived mesenchymal stem cells (MSCs) have unique properties that make them ideally suited for off-shelf clinical cell transplantation; they are easily extractable and are immune-privileged with multilineage potential [2]. Nevertheless, their therapeutic efficacy has been hindered by poor survival, retention, and engraftment of transplanted cells due to insufficient blood supply, poor nourishment of cells, and generation of free radicals [3]. Thus, it is reasonable to search for novel strategy promoting donor cell survival as well as optimizing their therapeutic effects.

Follistatin-like 1 (Fstl1), also known as TSC-36, is a secreted glycoprotein induced by transforming growth factor-β1 (TGF-β1) [4]. Previous literature has demonstrated that Fstl1 exerts cardiovascular-protective activities in ischemia and pathological cardiac hypertrophy models [5–7]. Recently, we reported that Fstl1 protects cardiomyoblasts from cell death through Akt and Smad1/5/9 signaling [8]. It is worthy of note that although cardiac Fstl1 level is markedly elevated in post-MI myocardium [6], retention and engraftment of donor cells in ischemic border zone are still very low [3]. We therefore propose that supplementation of intrinsic Fstl1 may further improve survival and engraftment of donor MSCs.

In the present study, we demonstrated that Fstl1 is a critical molecule determining the fate of implanted MSCs. Overexpression of Fstl1 in MSCs enhances their resistance to hypoxia and hence their potential in vivo lifespan. It is worthy of note that Fstl1-overexpressing MSCs improve post-MI cardiac function more effectively, with reduced fibrosis, inflammatory cell infiltration, and enhanced neovascularization in peri-infarct zones. In summary, our results support the promise of Fstl1 as an effective strategy to optimize stem cell-based therapy in tissue injury.

## Materials and methods

### Animals

C57BL/6J mice were obtained from the Experimental Animal Center of the Chinese Academy of Medicine Sciences of Soochow University. All animal protocols were approved based on the local ethics legislation with respect to animal experimentation.

### Culture and characterization of bone marrow MSCs

Bone marrow-derived MSCs from C57BL/6J mice (Cyagen Biosciences) were expanded in monolayer culture with mesenchymal stem cell growth medium (Cyagen Biosciences) supplemented with 10% fetal bovine serum at 37 °C until the cells reached 80% confluence as described previously [9]. The cells were then trypsinized and frozen in liquid nitrogen for later use. The enriched MSC population was characterized with antibodies against CD29, CD44, CD45, CD90, CD117, Sca-1, and their relative isotype controls on a flow cytometry (Millipore Guava easyCyte) as previously described [10].

### Lentivirus transduction of MSCs

The recombinant lentivirus for Fstl1 was purchased from GeneChem (GV320, China) and designated as LV-Fstl1. Order of the vector elements is Ubi-MCS-3FLAG-SV40-mCherry. The mCherry empty vector was used as a control and designated as LV-mCherry. MSCs infected with LV-Fstl1 or LV-mCherry at a multiplicity of infection of 10 were designated as MSCs-Fstl1 and MSCs-mCherry, respectively. Transduction efficiency was determined based on observation under fluorescent microscope 72 h after infection. Overexpression of Fstl1 in MSCs-Fstl1 was further confirmed by qRT-RCR and western blot analysis.

### Annexin V analysis and EdU incorporation assay

MSCs-mCherry and MSCs-Fstl1 were seeded in 6-well plates and incubated under hypoxic condition (94% N_2_, 5% CO_2_, and 1% O_2_) for 48 h. Cell death was measured by Annexin V-PE/7-AAD dead cell apoptosis kit (BD Pharmingen), and Annexin V-positive cells were quantified on a flow cytometry (Millipore Guava easyCyte) as described previously [11, 12]. Cells treated with 25 μM etoposide for 24 h were used as positive controls for apoptosis assay. Cell proliferation was assessed by click-it EdU flow cytometry kit (Life Technologies) according to the manufacturer’s instructions. Briefly, cells were incubated with 10 μM ethynyldeoxyuridine (EdU) for 2 h immediately after hypoxic treatment. Nuclear EdU was further marked by binding of azide group of click-it®Alexa Fluor 647 fluorophore to alkyne group of EdU. EdU incorporation was finally analyzed on a flow cytometry (Millipore Guava easyCyte).

### Tube formation assay

For the in vitro tube formation assay, 100 μL thawed Matrigel (BD Biosciences) was coated on 96-well plates at 37 °C for 1 h to allow the matrix to polymerize. Next, 2 × 10^4^ human umbilical vein endothelial cells (HUVECs) suspended in 50 μL endothelial growth medium-2 (EGM-2, Lonza) plus 50 μL MSC conditioned medium were seeded on the Matrigel and incubated at 37 °C for 12 h. Tube structures were inspected under an inverted light microscope. Tube length and branching per well were analyzed by ImageJ software as described previously [13, 14].

### MI and implantation of MSCs

Permanent MI was established by ligation of the left anterior descending coronary artery (LAD) in male C57BL/6J mice as previously described [15]. Briefly, animals were put under general anesthesia and ventilated by a rodent respirator. After a left thoracotomy between the third and fourth intercostal space, the left ventricle was exposed satisfactorily and LAD was ligated. Successful induction of MI was verified by a color change in the infarct region after ligation. Immediately after MI, 5 × 10^5^ cells suspended in 20 μL PBS were intramyocardially injected surrounding the infarct zones at two different sites. Finally, a thoracic incision was carefully closed, and the mice were allowed to recover. Whereas in the sham-operated group, the needle was passed around the artery without ligation.

### Animal study design

Mice were randomized into four groups: (1) sham, (2) MI/PBS, (3) MI/MSCs-mCherry, and (4) MI/MSCs-Fstl1. MSC engraftment was monitored by mCherry signal on post-therapy 1 day and by CM-DiI labeling on post-therapy 3 and 7 days (*n* = 3–10). Moreover, cardiac function was assessed by echocardiography on post-therapy 7 and 14 days (*n* = 6–10). Masson’s trichrome and immunofluorescent staining for vimentin, CD68, BS1 lectin, CD31, and α-SMA was performed on post-therapy 7 days to evaluate fibrosis, inflammatory cell infiltration, and neovascularization. Finally, total RNA from host myocardium proximal to transplanted cells was used to analyze the expression of fibrotic and inflammatory genes after cell transplantation (*n* = 3–4).

### Detection of MSCs recruitment

For detection of engrafted cells, MSCs were pre-labeled with 1 μg/mL chloromethylbenzamido (CM-DiI, Invitrogen) before cell therapy as described previously [16]. Alternate sets of serial vertical sections around the injection site were prepared and further monitored by fluorescent microscopy. Engrafted MSCs were also identified by co-localization of mCherry fluorescence (red) and immunofluorescent signal from anti-mCherry (green). Briefly, serial slides were sequentially incubated with rabbit anti-mCherry primary antibody (Abcam), FITC-conjugated anti-rabbit IgG (Senta Cruz), and DAPI-containing anti-fade medium and imaged under a fluorescent microscope.

### Echocardiographic measurements

Cardiac function of mice was evaluated by transthoracic echocardiography on Vevo 2100 system (VisualSonics, Canada) equipped with an 80-MHz probe as described previously [17]. All parameters were measured from M-mode recoding. Left ventricle ejection fraction (EF) and fractional shortening (FS) were automatically calculated by the echocardiography software using the following formulas: EF (%) = (LVID;d^3^ − LVID;s^3^)/LVID;d^3^ × 100% and FS (%) = (LVID;d − LVID;s)/LVID;d × 100%, respectively.

### Assessment of infarct size

Scar formation was analyzed using a Masson’s Trichrome Stain Kit (Sigma) as described previously [18]. In brief, hearts were collected and cut into frozen sections of 7 μm. Stained slides were photographed and quantified with ImageJ software. The percentage of infarct size was calculated as (fibrosis area/total LV area) × 100%.

### Immunofluorescent staining

Immunofluorescent staining was performed according to the standard protocol as previously reported [19]. Briefly, the heart sections were incubated with anti-vimentin, anti-CD68, anti-CD31 (Abcam), and anti-α-SMA (Santa Cruz) to detect fibroblasts, macrophages, and blood vessels. Then, FITC-conjugated anti-mouse IgG or anti-rabbit IgG was added and incubated for 1 h before observation. For detection of neovascularization, 1 mg/mL Griffonia (Bandeiraea) simplicifolia lectin 1 (BS1 lectin; Vector) was injected into the left ventricle via direct cardiac puncture 15 min before the sacrifice of mice. Slides were sequentially stained with goat anti-BS1 lectin antibody (Vector) and Alexa Fluor 488-conjugated anti-goat IgG (Jackson ImmunoResearch). Finally, the heart sections were mounted with DAPI-containing anti-fade medium and imaged.

### Reverse transcription PCR and quantitative RT-PCR

Total RNA was reverse transcribed to cDNA using the PrimeScript RT reagent Kit (Takara, Japan). qRT-PCR was performed using the SYBR Premix Ex Taq reaction mix (Takara, Japan) on a StepOne Plus real-time PCR system (Applied Biosystems) as previously reported [20]. The reaction conditions included 95 °C for 10 min and then 40 cycles of 95 °C for 15 s, 65 °C for 30 s, and 72 °C for 10 s. Expression of target genes was determined by comparative ΔΔCt method and *GAPDH* or *18S* was used as an internal control gene. The sequences of specific primer pairs are described below: *Fstl1*, 5′-TTATGATGGGCACTGCAA-3′ and 5′-ACTGCCTTTAGAGAACCAG-3′; *Fsp1*, 5′-AGGAGCTACTGACCAGGGAGCT-3′ and 5′-TCATTGTCCCTGTTGCTGTCC-3′; *α-SMA*, 5′-GCTGGTGATGATGCTCCCA-3′ and 5′-GCCCATTCCAACCATTACTCC-3′; *CTGF*, 5′-GGCCTCTTCTGCGATTTCG-3′ and 5′-GCAGCTTGACCCTTCTCGG-3′; *Col1a1*, 5′-CCAAGAAGACATCCCTGAAGTCA-3′ and 5′-TGCACGTCATCGCACACA-3′; *Fn1*, 5′-GTGTAGCACAACTTCCAATTACGAA-3′ and 5′-GGAATTTCCGCCTCGAGTCT-3′; *TNF-α*, 5′-AAACCACCAAGTGGAGGAGC-3′ and 5′-ACAAGGTACAACCCATCGGC-3′; *IL-6*, 5′-CGTGGACCTTCCAGGATGAG-3′ and 5′-CATCTCGGAGCCTGTAGTGC-3′; *IL-1β*, 5′-TGTAATGAAAGACGGCACAC-3′ and 5′-CTCCACTTTGCTCTTGACTTC-3′; *VEGF*, 5′-GCACATAGAGAGAATGAGCTT-3′ and 5′-CCCTCCGCTCTGAACAAGGCT-3′; *PDGF-BB*, 5′-TCCGGCTGCTGCAATAACC-3′ and 5′-GGCTTCTTTCGCACAATCTCAAT-3′; *IGF-1*, 5′-TCTGAGGAGGCTGGAGATGT-3′ and 5′-GTTCCGATGTTTTGCAGGTT-3′; *Ang-1*, 5′-ATCTTGATAACCGCAGCCAC-3′ and 5′-TGTCGGCACATACCTCTTGT-3′; *bFGF*, 5′-CCAGTTGGTATGTGGCACTG-3′ and 5′-CAGGGAAGGGTTTGACAAGA-3′; *GAPDH*, 5′-TGCCCAGAACATCATCCCT-3′ and 5′-GGTCCTCAGTGTAGCCCAAG-3′; and *18S*, 5′-GTAACCCGTTGAACCCCATT-3′ and 5′-CCATCCAATCGGTAGTAGCG-3′.

### Western blot analysis

Protein lysates were processed for western blot analysis following the standard protocol [21, 22]. The following primary antibodies were used to recognize the proteins: p-Akt (Ser473), Akt, p-GSK-3*β* (Ser9), GSK-3*β* (Cell Signaling Technology), Fstl1 (R&D), Vimentin (Abcam), α-SMA, and GAPDH (Santa Cruz). Immunoreactivity was detected by routine enzymatic chemiluminescence.

### Statistical analysis

Data were expressed as mean ± SEM. Statistical analysis was performed using ANOVA for multiple comparisons and two-tailed Student’s *t* test for comparisons between the two groups. *P* < 0.05 was considered statistically significant.

## Results

### Fstl1 expression declines dramatically in hypoxic MSCs

**Fig. 1:**
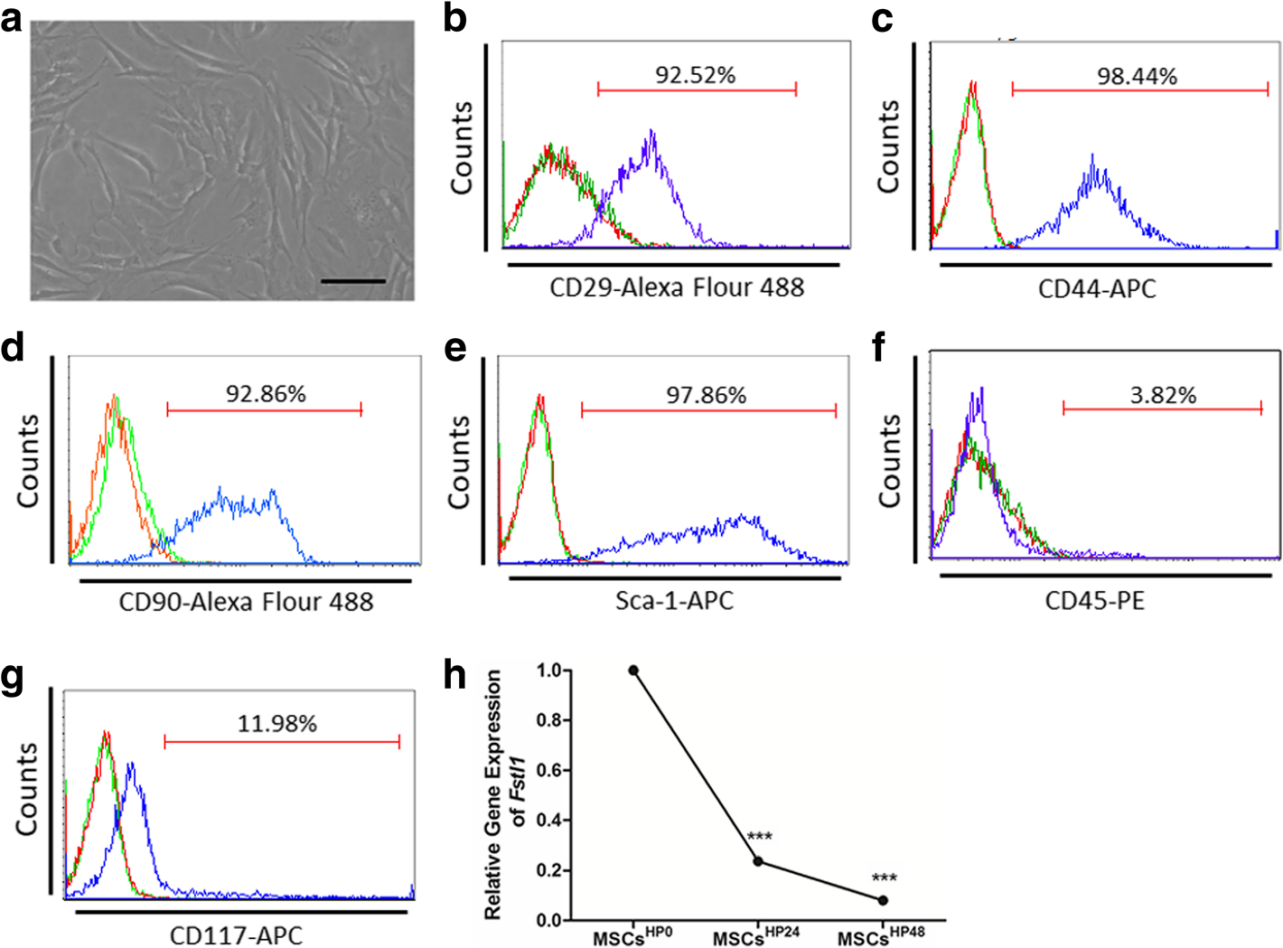
Characterization of bone marrow-derived MSCs and Fstl1 expression under hypoxic condition. **a** Morphology of murine bone marrow-derived MSCs in culture. These cells are adherent to bottom of plastic dishes. The majority of MSCs exhibit spindle or triangular shapes. Scale bar = 50 μm. **b**–**g** Most MSCs express mesenchymal stem cell marker CD29 (**b**), CD44 (**c**), CD90 (**d**), and Sca-1 (**e**), whereas most MSCs do not express hematopoietic marker CD45 (**f**) and progenitor marker CD117 (**g**). Unstained and isotype controls are indicated by red and green spectra, respectively. **h** qRT-PCR analysis of *Fstl1* mRNA levels in hypoxic MSCs (*n* = 5). ***P < 0.001, HP24 and HP48 versus HP0. HP, hypoxia

MSCs were isolated from the bone marrow of C57BL/6J mice. They are typically either spindle-shaped or triangular and adherent to plastic dishes (Fig. 1a). Cell surface markers of MSCs were further identified by flow cytometry (Fig. 1b–g). As expected, nearly all of the cells acquired in our study express typical mesenchymal stem cell markers: CD29 (92.52%), CD44 (98.44%), CD90 (92.86%), and Sca-1 (97.86%). Moreover, they are devoid of hematopoietic lineage marker CD45 (3.82%) and progenitor marker CD117 (11.98%), indicating characteristics of undifferentiated MSCs [23].

To unveil the potential role of Fstl1 in hypoxic MSCs, a time-course study was conducted on 0, 24, and 48 h to quantify its expression pattern. Notably, a rather dramatic decline of Fstl1 expression was observed in a time-dependent manner. As illustrated in Fig. 1h, Fstl1 expression in hypoxic MSCs is decreased to 23.71% at 24 h (*P* < 0.001 vs 0 h) and to 8.04% at 48 h (*P* < 0.001 vs 0 h).

### Efficient transduction of functionally active Fstl1 in MSCs

**Fig. 2:**
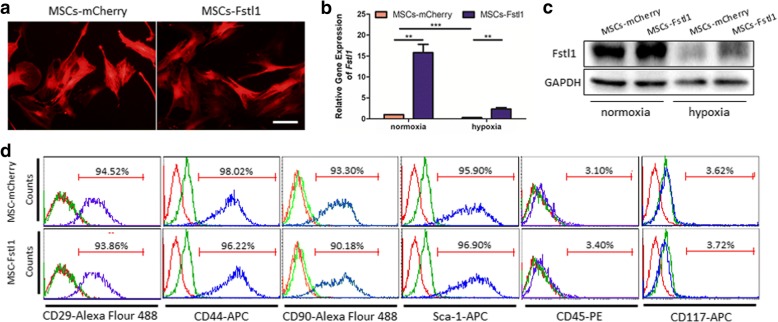
Construction of Fstl1-overexpressing MSCs. **a** Transduction efficiency of engineered MSCs evidenced by mCherry reporter expression. Scale bar = 50 μm. **b** *Fstl1* transcription in engineered MSCs was assessed by qRT-PCR (*n* = 4). **c** Protein level of Fstl1 in engineered MSCs was determined by western blot analysis. **d** Flow cytometry analysis of cell surface antigens of genetically modified MSCs. Unstained and isotype controls are indicated by red and green spectra, respectively. **P < 0.01; ***P < 0.001

To further identify whether restoration of intrinsic Fstl1 is able to exert potential benefits on MSCs behavior against hypoxic challenge, we overexpressed Fstl1 by gene modification. A recombinant lentiviral vector expressing the transgene Fstl1 (LV-Fstl1) was constructed, and a blank vector containing no transgene (LV-mCherry) was used as a control. All lentiviral vectors were designed to carry a mCherry reporter. For lentiviral infection, MSCs were exposed to viral supernatant at a multiplicity of infection (MOI) of 10 for 24 h. After infection, most MSCs express mCherry fluorescence (red), suggesting successful lentiviral packaging and infection (Fig. 2a). Cell morphology remains unchanged following lentiviral transduction; therefore, infected MSCs (MSCs-mCherry and MSCs-Fstl1) were used in future experiments. Efficient transduction of MSCs was further confirmed by qRT-PCR and western blot, showing increased mRNA and protein level of Fstl1 in MSCs-Fstl1 (Fig. 2b, c). Besides, to mimic the ischemic microenvironment of infarcted hearts, both MSCs were maintained under hypoxic conditions for 48 h. Though Fstl1 expression in both MSCs declines dramatically following hypoxic treatment, its level in MSCs-Fstl1 remains higher than that of MSCs-mCherry (Fig. 2b, c). Finally, MSCs-Fstl1 showed similar mesenchymal marker expression pattern with MSCs-mCherry. They are both positive for CD29, CD44, CD90, and Sca-1 and negative for CD45 and CD117 (Fig. 2d).

### Fstl1 enhances survival and proliferation of MSCs against hypoxic challenge

**Fig. 3:**
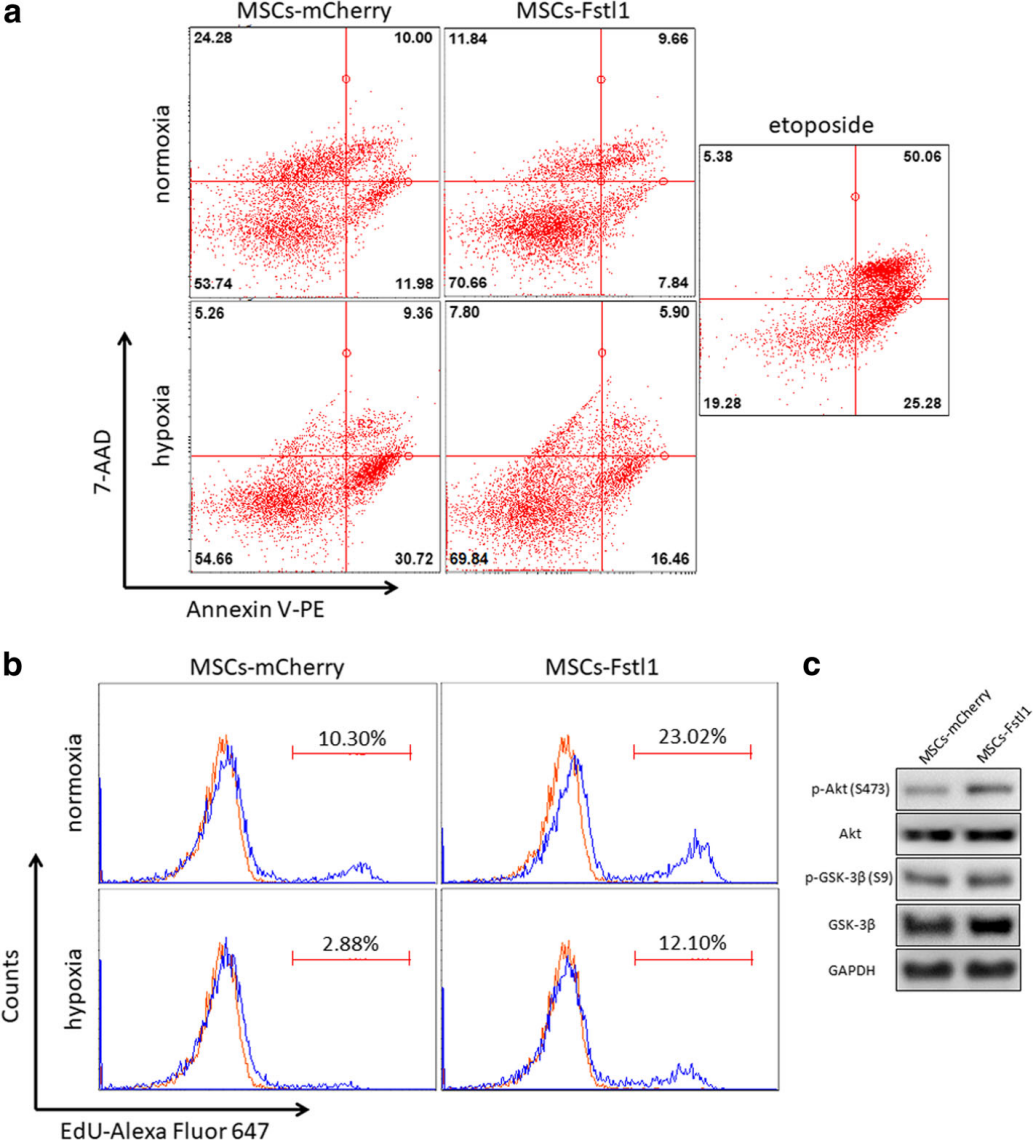
Fstl1 facilitates survival and proliferation of MSCs under hypoxic condition. Engineered MSCs were challenged with hypoxia for 48 h. **a** Flow cytometry of Annexin V-PE/7-AAD staining. Etoposide-treated cells were used as positive controls. **b** Cell proliferation detected using EdU incorporation assay. Unstained control is indicated by red spectra. **c** Western blot analysis of p-Akt (Ser473), Akt, p-GSK-3*β* (Ser9), and GSK-3β in hypoxic MSCs

To investigate the potential role of Fstl1 on MSCs behavior, we examined in vitro survival and proliferation of MSCs-Fstl1 against hypoxic challenge. We first performed flow cytometry of Annexin V-PE/7-AAD to evaluate cell death of these genetically modified MSCs under both normoxic and hypoxic conditions. As shown in Fig. 3a, hypoxia triggers an elevation in early (Annexin V^+^ 7-AAD^−^) and late (Annexin V^+^ 7-AAD^+^) apoptosis in both cells. Importantly, the percentage of both early and late apoptotic cells in MSCs-Fstl1 is much lower compared to that in MSCs-mCherry under both conditions, suggesting that MSCs-Fstl1 has superior resistance to hypoxia-induced cell death. In addition, we also performed flow cytometry of ethynyldeoxyuridine (EdU) to examine the proliferation of these genetically modified MSCs under both normoxic and hypoxic conditions. Specifically, both MSCs exhibit attenuated proliferative ability after hypoxic treatment. Nonetheless, the percentage of EdU-positive proliferating cells in MSCs-Fstl1 is still higher than that in MSCs-mCherry under both conditions, indicating intrinsic Fstl1 benefits MSCs proliferation (Fig. 3b). We and other scholars have provided evidence that the pro-survival Akt signaling pathway is directly regulated by Fstl1 [8]. To further elucidate how intrinsic Fstl1 rescues hypoxic MSCs dysfunction, we collected both MSCs 48 h after hypoxia and measured phosphorylation of Akt-Ser473 (p-Akt) and its downstream target GSK3β-Ser9 (p-GSK3β). As expected, both p-Akt and p-GSK3β are elevated in hypoxic MSCs-Fstl1, indicating activation of Akt/GSK-3β signaling may be responsible for its cell death resistance (Fig. 3c). These in vitro results demonstrate that intrinsic Fstl1 directly alters MSCs behavior, and MSCs-Fstl1 are more tolerant than MSCs-mCherry to the hypoxia-induced challenge.

### Fstl1 promotes retention of engrafted MSCs in ischemic myocardium

**Fig. 4:**
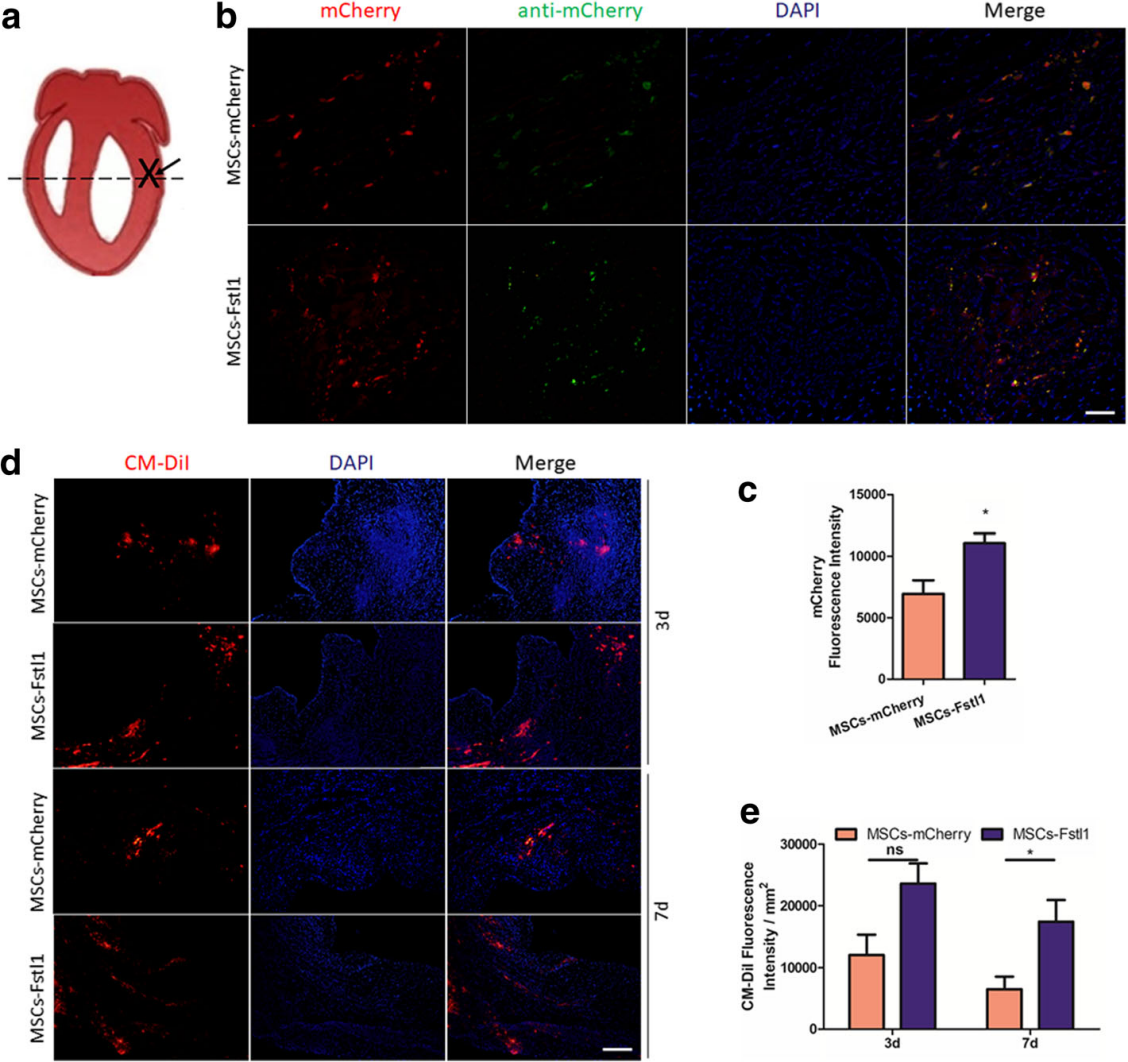
Localization of engrafted MSCs in infarcted hearts. **a** Heart schematic pinpointing the location of ligation (cross), injection (arrow), and sampling (dashed line). **b** Engraftment of genetically modified MSCs indicated by co-localization of mCherry fluorescence (red) and immunofluorescent signal against mCherry (green) on post-injection 1 day. Scale bar = 50 μm. **c** Quantification of cell engraftment by mCherry fluorescence (red) (*n* = 3–4). **d** Representative images of surviving MSCs indicated by CM-Dil staining on post-therapy 3 and 7 days. Scale bar = 200 μm. **e** Quantification of cell engraftment by CM-Dil signal (*n* = 5–10). *P < 0.05. CM-Dil, chloromethylbenzamido

One of the challenging barriers against MSCs therapy in MI is low engraftment of donor cells due to limited survival rate in the hypoxic environment [24]. We therefore assessed survival and retention of either MSCs-mCherry or MSCs-Fstl1 in ischemic myocardium (Fig. 4a). Transplanted MSCs were identified by co-localization of mCherry fluorescence (red) and immunofluorescent signal with anti-mCherry plus FITC-conjugated anti-IgG (green) on post-therapy 1d. As indicated in Fig. 4b, most mCherry and FITC fluorescence are co-localized, and a large number of mCherry-positive cells were found dispersed in the injection region. It is important to note, compared to MI/MSCs-mCherry hearts, obviously, more mCherry-positive cells are present in MI/MSCs-Fstl1 hearts (*P* < 0.05, Fig. 4c). Moreover, to further track the injected cells in vivo, both MSCs were pre-labeled with CM-DiI before subjected for transplantation. As illustrated in Fig. 4d, clusters of CM-DiI signals were observed in the ischemic border zone after cell transplantation. More importantly, MI/MSCs-Fstl1 group possesses even more CM-Dil signals compared with MI/MSCs-mCherry group on post-therapy 7 days (*P* < 0.05, Fig. 4e). Finally, although MSCs-Fstl1 exhibit better retention, total Fstl1 level in MSCs-Fstl1-treated ischemic myocardium remains similar to that in MSCs-mCherry-treated tissue (Additional file 1: Figure S1). Collectively, these results indicate that Fstl1-overexpressing MSCs exhibit better retention in ischemic myocardium.

### MSCs-Fstl1 implantation preserves post-MI heart function more effectively

**Fig. 5:**
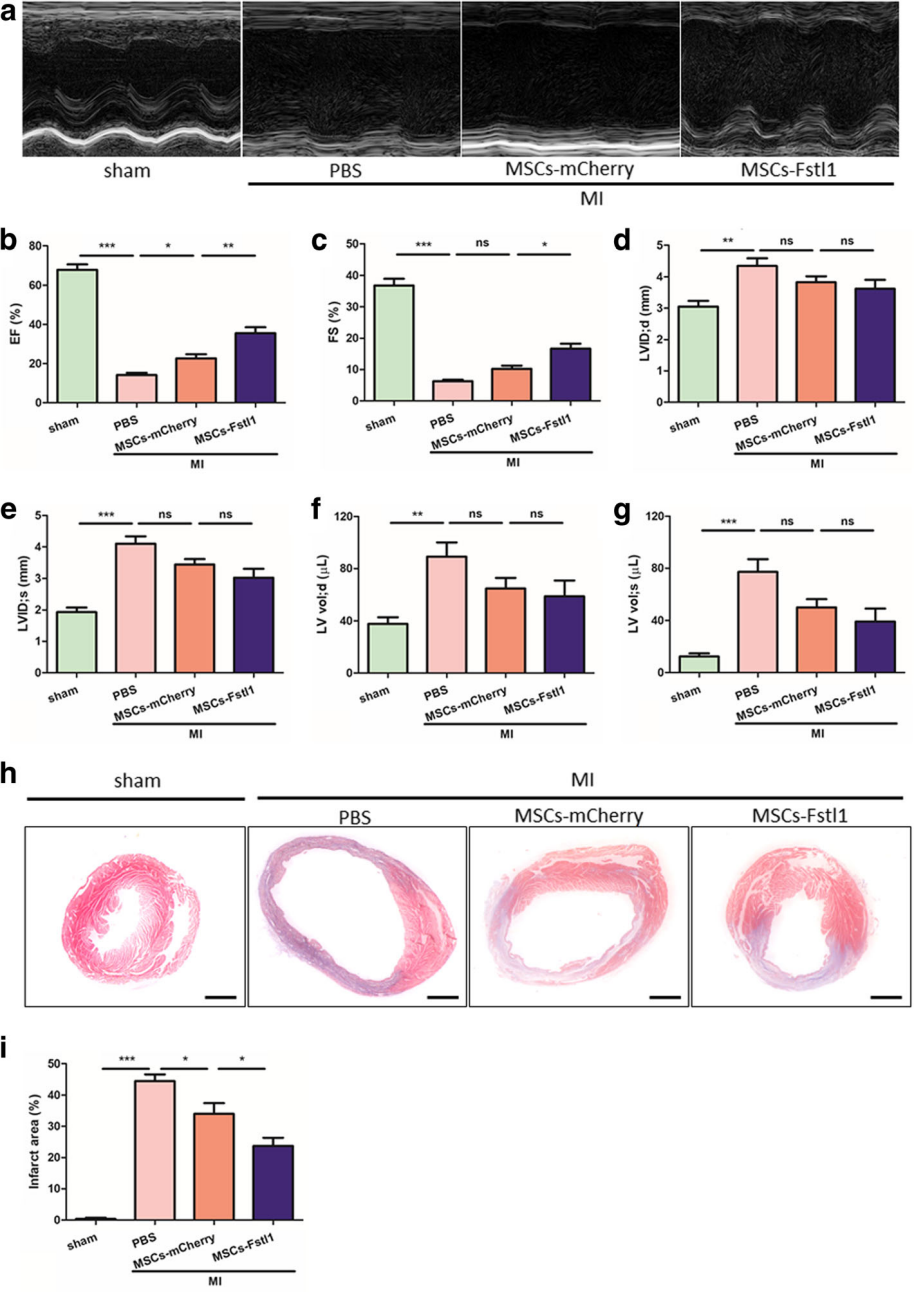
Enhanced post-MI cardiac function and limited scar formation after MSCs-Fstl1 transplantation. **a** Representative echocardiographic images on post-therapy 7 days. EF (**b**), FS (**c**), LVID;d (**d**), LVID;s (**e**), LV vol;d (**f**), and LV vol;s (**g**) were analyzed, respectively (*n* = 7–10). Macroscopic view (**h**) and statistical analysis (**i**) of Masson’s trichrome staining on post-therapy 7 days. Scale bar = 1 mm. (*n* = 5). *P < 0.05; **P < 0.01; ***P < 0.001. EF, ejection fraction; FS, fractional shortening; ns, not significant

We assessed in vivo therapeutic effects of MSCs-Fstl1 on a mouse MI model by echocardiography (Fig. 5a). As illustrated in Fig. 5b, ejection fraction (EF) following MI declines dramatically (14.18 ± 1.12% versus 67.88 ± 2.78% in the sham group, *P* < 0.001). Importantly, MI-induced reduction in EF is partially reversed by MSCs-mCherry treatment (22.73 ± 2.07% versus 14.18 ± 1.12% in the MI/PBS group, *P* < 0.05) and is further recovered with greater significance following MSCs-Fstl1 transplantation (35.55 ± 3.00% versus 22.73 ± 2.07% in the MI/MSCs-mCherry group, *P* < 0.05). Moreover, fractional shortening (FS, %) confirms a similar tendency to EF (Fig. 5c). Additionally, compared to the sham group, left ventricular dimensions and volumes are all enlarged following MI (0.42-, 1.12-, 1.36-, and 5.24-fold enlargement in LVID;d, LVID;s, LV vol;d, and LV vol;s, respectively), exhibiting characteristics of MI (Fig. 5d–g). Nonetheless, wall thickness and LV mass remain unchanged by MSCs-Fstl1 treatment (Additional file 2: Figure S2). Importantly, improved post-MI EF and FS by MSCs-Fstl1 treatment can also be long-term monitored (Additional file 3: Figure S3). Finally, the infarct area was assessed using Masson trichrome staining (Fig. 5h–i). MI-induced scar formation is partially reversed by MSCs-mCherry treatment (34.04 ± 3.42% versus 44.50 ± 2.16% in the MI/PBS group, *P* < 0.05) and is further recovered with greater significance following MSCs-Fstl1 transplantation (23.71 ± 2.62% versus 34.04 ± 3.42% in the MI/MSCs-mCherry group, *P* < 0.05). Collectively, MSCs-Fstl1 implantation effectively ameliorates post-MI cardiac dysfunction and scar formation.

### MSCs-Fstl1 ameliorates fibroblasts accumulation and ECM production in ischemic myocardium

**Fig. 6:**
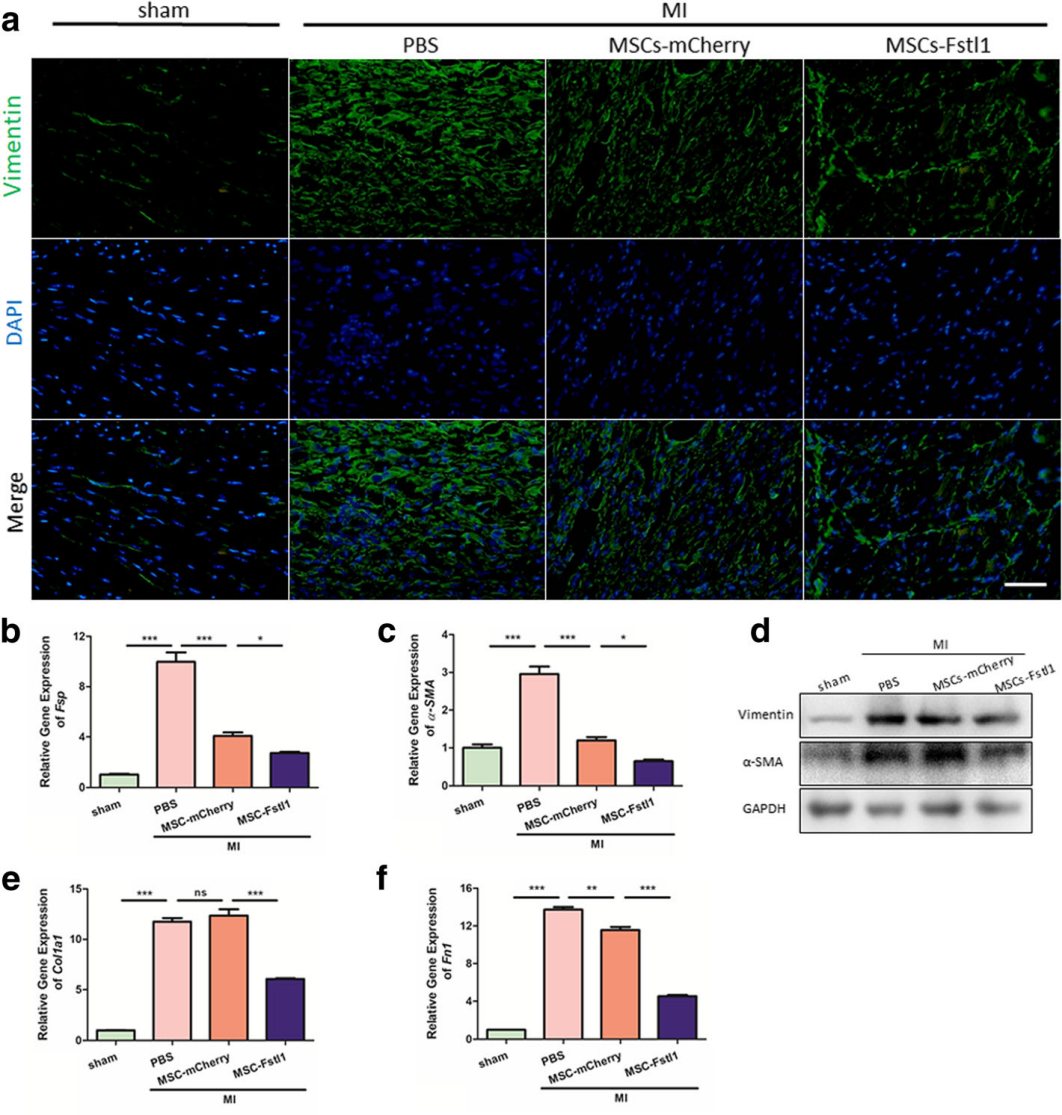
Fibroblast accumulation and ECM deposition are ameliorated in MSCs-Fstl1-treated ischemic myocardium. **a** Representative images of vimentin staining within the infarct border zone on post-therapy 7 days. Scale bar = 50 μm. qRT-PCR analysis of *Fsp-1* (**b**), *α-SMA* (**c**), *Col1a1* (**e**), and *Fn1* (**f**) in peri-infarct myocardium on post-therapy 7 days (*n* = 3–4). **d** Western blot analysis of vimentin and *α*-SMA in ischemic myocardium on post-therapy 7 days. *P < 0.05; **P < 0.01; ***P < 0.001. MI, myocardial infarction; *Fsp-1*, fibroblast-specific protein-1; α*-SMA*, α-smooth muscle actin; *Col1a1*, type I collagen; *Fn1*, fibronectin; ns, not significant

During the late phase of MI, damaged tissue is replaced with a fibrotic scar with cross-linked collagen produced by fibroblasts and myofibroblasts. During this process, the healing heart undergoes profound changes in ventricular geometry, biomechanics, and function. Although the initial reparative fibrosis is beneficial for preventing rupture of the ventricular wall, an exaggerated fibrotic response outside the injured area is detrimental as it leads to cardiac remodeling and eventually heart failure [25]. Immunostaining for vimentin (a fibroblast marker) exhibited least fibroblasts accumulation in MSCs-Fstl1-treated peri-infarct zone (Fig. 6a). Moreover, MI markedly elevates mRNA levels of fibroblast-specific protein-1 (*Fsp-1*, a marker for fibroblasts) and α-smooth muscle actin (*α-SMA*, a marker for myofibroblasts). In contrast, MSCs-mCherry administration significantly attenuates MI-increased levels of cardiac *Fsp-1* by 58.39% (*P* < 0.001) and *α-SMA* by 59.16% (*P* < 0.001). Notably, MSCs-Fstl1 transplantation further diminishes *Fsp-1* and *α-SMA* levels by 33.63% (*P* < 0.05) and 40.27% (*P* < 0.05), respectively (Fig. 6b, c). Western blot analysis confirmed a similar tendency of these two fibrotic markers (Fig. 6d). Additionally, subsequent ECM production is also attenuated by MSCs-Fstl1 treatment, as evidenced by lowest transcription for type I collagen (*Col1a1*) and fibronectin (*Fn1*) in peri-infarcted zones (Fig. 6e, f). Finally, both MSCs-mCherry and MSCs-Fstl1 treatment dramatically abolish expression of connective tissue growth factor (*CTGF*, a pro-fibrogenic cytokine) in ischemic myocardium (Additional file 4: Figure S4). In summary, we observed attenuated fibroblasts accumulation and subsequent ECM deposition in MSCs-Fstl1-treated myocardium.

### MSCs-Fstl1 treatment reduces myocardial infiltration of inflammatory cells in peri-infarct zones

**Fig. 7:**
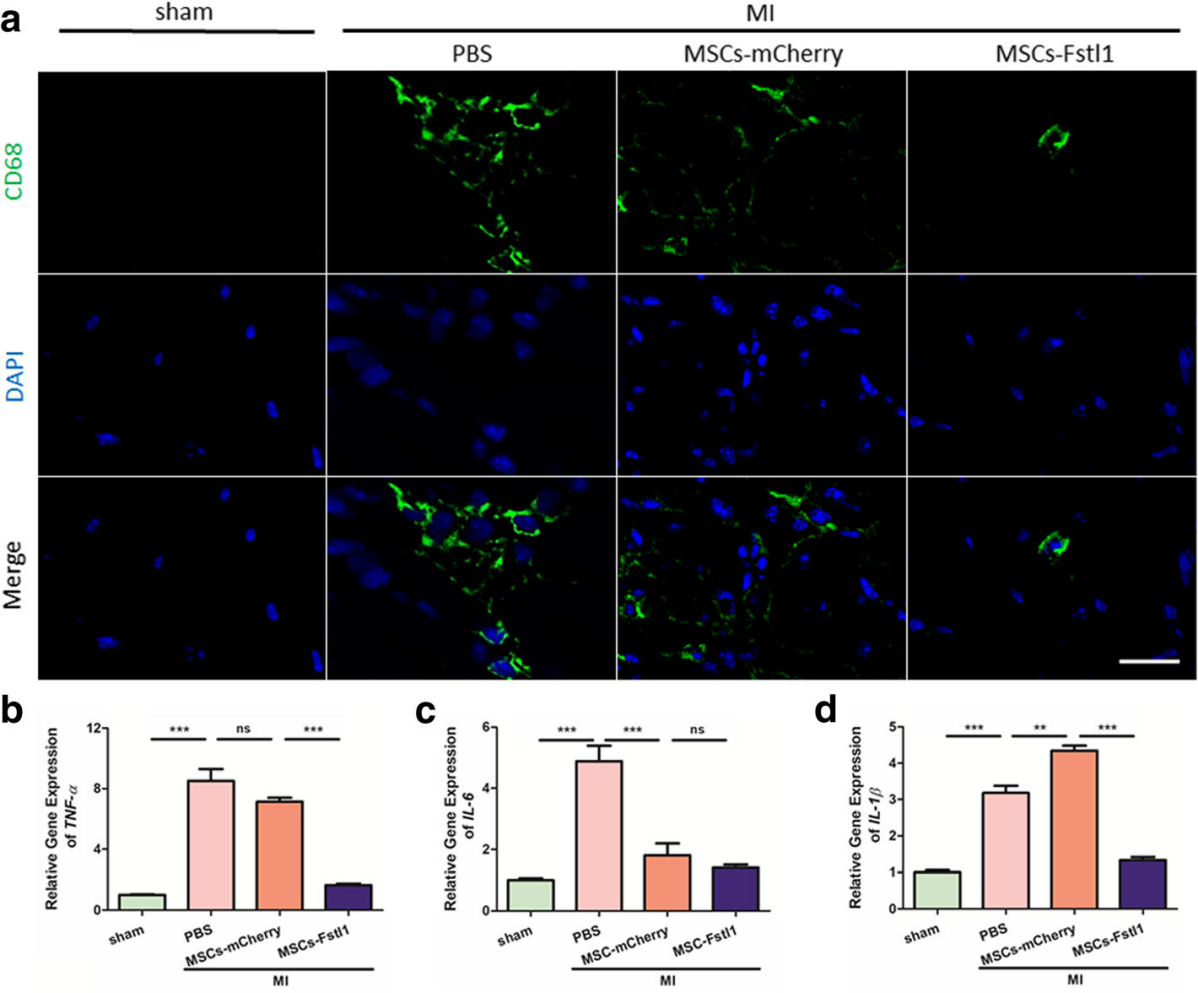
MSCs-Fstl1 treatment abrogates myocardial infiltration of inflammatory cells in peri-infarct zones. **a** Representative images of CD68-positive macrophage infiltration within the infarct border zone on post-therapy 7 days. Scale bar = 20 μm. **b**–**d** qRT-PCR analysis of *TNF-α* (**b**), *IL-6* (**c**), and *IL-1β* (**d**) in ischemic myocardium on post-therapy 7 days (*n* = 3–4). **P < 0.01; ***P < 0.001. *TNF-α*, tumor necrosis factor-α; *IL-6*, interleukin-6; *IL-1β*, interleukin-1β; ns, not significant

Acute MI elicits a robust innate immune response [26] and mobilizes a large population of macrophages to the myocardium [27]. As a central cellular protagonist in innate immunity, excessive inflammation triggered by macrophages can exacerbate post-MI cardiac dysfunction [28]. To evaluate the extent and severity of myocardial inflammation, the heart sections were stained with anti-CD68 antibody to identify infiltrated macrophages in peri-infarct myocardium 7 days post-therapy. As shown in Fig. 7a, transplantation of MSCs-Fstl1 impairs CD68-positive macrophage infiltration in peri-infarct zones most effectively. Similar results were further confirmed by measurement of cardiac *TNF-α*, *IL-6*, and *IL-1β* in different groups. As illustrated in Fig. 7b–d, MI significantly increases cardiac *TNF-α* by 7.66-fold (*P* < 0.001), *IL-6* by 3.88-fold (*P* < 0.001), and *IL-1β* by 2.18-fold (*P* < 0.001) compared with that in sham control. Nonetheless, MSCs-Fstl1 implantation diminishes cardiac *TNF-α* by 76.87% (*P* < 0.001) and *IL-1β* by 69.65% (*P* < 0.001), compared with that in the MI/MSCs-mCherry group. Though local inflammation extent differs among four groups, serum levels of TNF-α and IL-1β remain unexpectedly similar on post-therapy 7 days (Additional file 5: Figure S5). In summary, we observed attenuated macrophage infiltration and inflammatory response in MSCs-Fstl1-treated ischemic hearts.

Macrophages play both beneficial and detrimental roles in the wound healing process after MI [29, 30]. It is well documented that polarized macrophages can be classified mainly into two different phenotypes: proinflammatory (M1) and anti-inflammatory (M2). To further validate whether altered macrophage polarization is involved in MSCs-Fstl1-treated ischemic myocardium, we measured mRNA levels of both M1 (iNOS, CD80) and M2 (Argnase-1, CD206) markers on post-therapy 7 days. As illustrated in Additional file 6: Figure S6, expression of iNOS, CD80, Argnase-1, and CD206 all remain unchanged between MI/MSCs-mCherry and MI/MSCs-Fstl1, suggesting that MSCs-Fstl1 regulates post-MI cardiac remodeling via a mechanism that may be distinct from macrophage polarization.

### MSCs-Fstl1 transplantation accelerates neovascularization in peri-infarct region

**Fig. 8:**
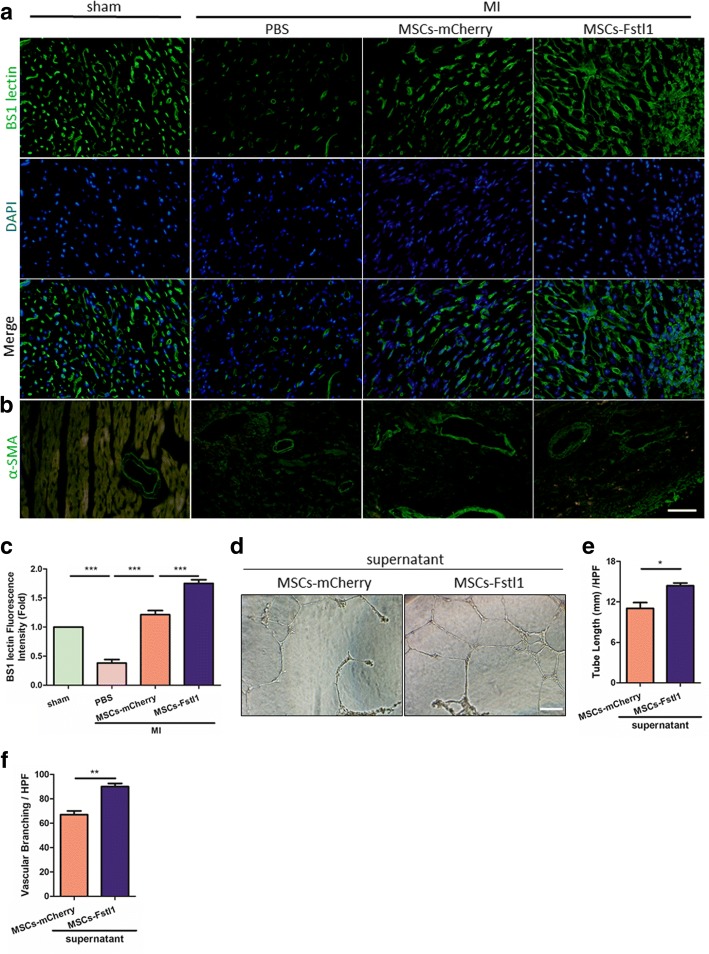
Enhanced neovascularization in MSCs-Fstl1-treated peri-infarct zones. Representative images of BS1 lectin (**a**) and α-SMA (**b**) staining within the infarct border zone on post-therapy 7 days. Scale bar = 50 μm. **c** Vascular density determined by BS1 lectin signal (*n* = 4). **d**–**f** HUVECs were incubated with a mixture of EGM-2 and MSCs conditioned medium as illustrated (1:1) for 12 h on Matrigel. **d** Representative images of the capillary network are shown. Scale bar = 100 μm. Quantification of tube length (**e**) and vascular branching (**f**) per high-power fields (HPF) were performed (*n* = 3). *P < 0.05; **P < 0.01; ***P < 0.001. α-SMA, α-smooth muscle actin; HPF, high-power fields

Development of great vessels and capillaries, referred to as neovascularization, is a crucial process in post-MI recovery [31]. More importantly, an increasing volume of literature has reported that Fstl1 participates in the determination of vessel fate [5, 32]. We therefore hypothesized that forced secretion of Fstl1 by its gene-modified MSCs may help accelerate neovascularization during post-MI recovery. For detection of capillaries, BS1 lectin was infused into the left ventricle via direct cardiac puncture before mice were sacrificed. Capillary density was revealed through subsequent immunofluorescent staining with anti-BS1 lectin (Fig. 8a). Compared with MI/PBS group, transplantation of MSCs-mCherry or MSCs-Fstl1 achieves obviously richer capillary network in the peri-infarct region, and even more condensed capillaries are observed after MSCs-Fstl1 implantation (Fig. 8c). A similar tendency of neovascularization was also observed with CD31 immunofluorescent signal (Additional file 7: Figure S7). Additionally, the blood vessels in the peri-infarct area were also evaluated by α-SMA (a marker for smooth muscle cells) staining. As illustrated in Fig. 8b, blood vessel density is greatly increased in the MI/MSCs-mCherry and MI/MSCs-Fstl1 groups compared to that in the MI/PBS group on post-therapy 7 days. In order to evaluate the paracrine angiogenic potential of MSCs-Fstl1, HUVECs were incubated with a mixture of EGM-2 and conditioned medium from indicated MSCs on Matrigel-precoated wells. As illustrated in Fig. 8d, HUVECs maintained in conditioned medium from MSCs-mCherry exhibit some tube-like structures and half-full cellular networks. Intriguingly, MSCs-Fstl1 culture medium facilitates HUVECs to form full and much denser cellular networks. The cumulative capillary tube length and branching in MSCs-Fstl1 supernatant group increased by 30.31% and 34.33% compared to that with MSCs-mCherry culture medium (Fig. 8e, f). Finally, the expression of several pro-angiogenic paracrine cytokines, including *VEGF*, *PDGF-BB*, *IGF-1*, *Ang-1*, and *bFGF*, remain unchanged between MSCs-mCherry and MSCs-Fstl1 under hypoxic conditions, indicating genetic overexpression of Fstl1 in MSCs does not alter the expression pattern of most pro-angiogenic paracrine factors (Additional file 8: Figure S8). In summary, we observed richest network of capillaries and blood vessels in MSCs-Fstl1-treated host myocardium and the pro-angiogenetic potential of MSCs-Fstl1 is probably mediated through a paracrine mechanism.

## Discussion

Stem cell therapy for the repair of damaged myocardium has evolved into a promising treatment for ischemic heart diseases. MSC-based therapy, originating from BM-, adipose tissue-, or umbilical cord-cells, continues to gain consent and appeal, because of the large body of preclinical evidence supporting higher paracrine cardio-reparative potential [33]. MSC-mediated tissue repair has been reported to dampen inflammation as well as promote neovascularization in ischemic myocardium through paracrine mechanisms [34, 35]. However, poor survival of donor cells and failure of their subsequent engraftment occur within the first days after delivery, posing a significant challenge in the field [36]. Finding a new method to improve survival and engraftment of MSCs in the injured myocardium is therefore imperative to optimize their therapeutic application.

There are several major findings in this study. First, intrinsic Fstl1 expression in MSCs declines dramatically following hypoxia. Second, MSCs-Fstl1 is more tolerant than MSCs-mCherry to the hypoxic challenge. Third, compared with MSCs-mCherry, MSCs-Fstl1 exhibits better retention and pro-angiogenic capacity following MI. Finally, MSCs-Fstl1-mediated cardiac repair is associated with reduced post-MI fibroblasts accumulation, ECM deposition, and inflammatory cell infiltration.

As a pro-survival cellular factor, we and others have demonstrated that Fstl1 inhibits cell death of cardiomyocytes [6], H9c2 [8], and endothelial cells [5] under various conditions. We validated here that restoration of intrinsic Fstl1 in MSCs improves their behavior and subsequent in vivo engraftment. In accordance with our observations, Holmfeldt et al. identified Fstl1 as one of the 17 novel regulators of hematopoietic stem cell repopulation [37]. Additionally, periodic secretion of Fstl1 by feeder cells also facilitates telomere maintenance and long-term self-renewal of mESCs by enhancing sporadic Zscan4 expression [38]. Importantly, we also validated activation of the pro-survival Akt/GSK-3β signaling in hypoxic MSCs-Fstl1, consistent with the previous report that retroviral-mediated overexpression of Akt1 also enhances survival of MSCs in an ischemic setting [39].

Various, seemingly contradictory effects of Fstl1 on cell proliferation and growth have been reported. On one hand, the results of our study demonstrated that Fstl1 promotes MSCs proliferation under hypoxic conditions (12.30% versus 3.00% in MSCs-mCherry). Similarly, restoration of the epicardial Fstl1 also enhances proliferation of immature cardiomyocytes, and consequently, activates regeneration of the adult mammalian heart, and reverses post-MI remodeling [40]. On the other hand, Fstl1 seems to inhibit pathological cell proliferation and therefore benefit proliferative diseases. For example, Fstl1 inhibits proliferation and migration of vascular smooth muscle cells [41] and, consequently, attenuates neointimal formation in response to arterial injury through an AMPK-dependent mechanism [42]. Moreover, Fstl1 has also been identified as a tumor suppressor in ovarian and endometrial tumors partially through inhibition of cell proliferation, migration, and invasion [43].

## Conclusions

In conclusion, our current data demonstrated a favorable role of Fstl1 in stem cell-based therapy for experimental myocardial infarction. Fstl1 enhances survival and engraftment of transplanted cells, thereby promoting neovascularization as well as alleviating myocardial ECM deposition and inflammatory cell infiltration in ischemic hearts. Our data support the promise of Fstl1-overexpressing MSCs as a novel strategy to improve MSCs-based therapy for ischemic diseases.

## Additional files

Additional file 1: **Figure S1.** Murine heart tissue around the injection site was collected 1 day after myocardial delivery of indicated MSCs and expression of Fstl1 was determined by qRT-PCR (*n* = 4). ns not significant. (PDF 115 kb)

Additional file 2: **Figure S2.** Echocardiographic parameters regarding wall thickness and LV mass of post-MI hearts. IVS;d (a), IVS;s (b), LVPW;d (c), LVPW;s (d), and LV mass (e) were analyzed on post-therapy 7 days respectively (*n* = 7–10). **P < 0.01. IVS;d interventricular septum in diastole, IVS;s interventricular septum in systole, LVPW;d left ventricular posterior wall in diastole, LVPW;s left ventricular posterior wall in systole, LV mass left ventricular mass, ns not significant. (PDF 197 kb)

Additional file 3: **Figure S3.** Changing tendency of post-MI EF (a) and FS (b) on indicated time points were determined (*n* = 6–7). *MI/MSCs-Fstl1 vs MI/MSCs-mCherry, *P* < 0.05; **MI/MSCs-Fstl1 vs MI/MSCs-mCherry, *P* < 0.01; ^&&&^MI/PBS vs sham, *P* < 0.001. EF ejection fraction, FS fractional shortening. (PDF 164 kb)

Additional file 4: **Figure S4.** qRT-PCR analysis of *CTGF* in peri-infarct myocardium on post-therapy 7 days (*n* = 4). ***P < 0.001. *CTGF* connective tissue growth factor, ns not significant. (PDF 115 kb)

Additional file 5: **Figure S5.** Serum TNF-α (a) and IL-1β (b) on post-therapy 7 days was determined by ELISA (*n* = 4). TNF-α tumor necrosis factor-α, IL-1β interleukin-1β, ns not significant. (PDF 131 kb)

Additional file 6: **Figure S6.** qRT-PCR analysis of M1 (*iNOS*, *CD80*) and M2 markers (*Argnase-1*, *CD206*) in peri-infarct myocardium on post-therapy 7 days (*n* = 3–4). (PDF 128 kb)

Additional file 7: **Figure S7.** Representative images (a) and quantification (b) of CD31 staining within the infarct border zone on post-therapy 7 days. Scale bar = 50 μm. (*n* = 4–5). (PDF 142 kb)

Additional file 8: **Figure S8.** qRT-PCR analysis of *VEGF*, *PDGF-BB*, *IGF-1*, *Ang-1*, and *bFGF* in hypoxic MSCs (*n* = 3–4). **P < 0.01. *VEGF* vascular endothelial growth factor, *PDGF-BB* platelet-derived growth factor-BB, *IGF-1* insulin-like growth factor 1, *Ang-1* angiopoietin-1, *bFGF* fibroblast growth factor-basic, ns not significant. (PDF 133 kb)

## Abbreviations

CM-Dil: Chloromethylbenzamido
Col1a1: Type I collagen
EdU: Ethynyldeoxyuridine
EF: Ejection fraction
Fn1: Fibronectin
FS: Fractional shortening
Fsp-1: Fibroblast-specific protein-1
Fstl1: Follistatin-like 1
IL-1β: Interleukin-1β
LV vol;d: Left ventricular volume in diastole
LV vol;s: Left ventricular volume in systole
LVID;d: Left ventricular internal diameter in diastole
LVID;s: Left ventricular internal diameter in systole
MI: Myocardial infarction
MOI: Multiplicity of infection
MSCs: Mesenchymal stem cells
PBS: Phosphate-buffered saline
PCR: Polymerase chain reaction
qRT-PCR: Quantitative RT-PCR
TGF-β: Transforming growth factor-β
α-SMA: α-Smooth muscle actin

## References

[1] Garbern JC, Lee RT (2013). Cardiac stem cell therapy and the promise of heart regeneration. Cell Stem Cell.

[2] Williams AR, Hare JM (2011). Mesenchymal stem cells: biology, pathophysiology, translational findings, and therapeutic implications for cardiac disease. Circ Res.

[3] Bartunek J, Behfar A, Dolatabadi D (2013). Cardiopoietic stem cell therapy in heart failure: the C-CURE (Cardiopoietic stem cell therapy in heart failURE) multicenter randomized trial with lineage-specified biologics. J Am Coll Cardiol.

[4] Shibanuma M, Ji M, Mita A (1993). Cloning from a mouse osteoblastic cell line of a set of transforming-growth-factor-β1-regulated genes, one of which seems to encode a follistatin-related polypeptide. Eur J Biochem.

[5] Ouchi N, Oshima Y, Ohashi K (2008). Follistatin-like 1, a secreted muscle protein, promotes endothelial cell function and revascularization in ischemic tissue through a nitric-oxide synthase-dependent mechanism. J Biol Chem.

[6] Oshima Y, Ouchi N, Sato K (2008). Follistatin-like 1 is an Akt-regulated cardioprotective factor that is secreted by the heart. Circulation.

[7] Shimano M, Ouchi N, Nakamura K (2011). Cardiac myocyte follistatin-like 1 functions to attenuate hypertrophy following pressure overload. Proc Natl Acad Sci U S A.

[8] Chen W, Xia J, Hu P (2016). Follistatin-like 1 protects cardiomyoblasts from injury induced by sodium nitroprusside through modulating Akt and Smad1/5/9 signaling. Biochem Biophys Res Commun.

[9] He J, Teng X, Yu Y (2013). Injection of Sca-1+/CD45+/CD31+ mouse bone mesenchymal stromal-like cells improves cardiac function in a mouse myocardial infarct model. Differentiation.

[10] Chen W, Wang S, Xia J (2016). Protein phosphatase 2A plays an important role in migration of bone marrow stroma cells. Mol Cell Biochem.

[11] Chen W, Gu P, Jiang X (2011). Protein phosphatase 2A catalytic subunit α (PP2Acα) maintains survival of committed erythroid cells in fetal liver erythropoiesis through the STAT5 pathway. Am J Pathol.

[12] Wu J, Dong Y, Teng X (2015). Follistatin-like 1 attenuates differentiation and survival of erythroid cells through Smad2/3 signaling. Biochem Biophys Res Commun.

[13] Hou J, Wang L, Wu Q (2018). Long noncoding RNA H19 upregulates vascular endothelial growth factor A to enhance mesenchymal stem cells survival and angiogenic capacity by inhibiting miR-199a-5p. Stem Cell Res Ther.

[14] Hou J, Zhong T, Guo T (2017). Apelin promotes mesenchymal stem cells survival and vascularization under hypoxic-ischemic condition in vitro involving the upregulation of vascular endothelial growth factor. Exp Mol Pathol.

[15] Teng X, Chen L, Chen W (2015). Mesenchymal stem cell-derived exosomes improve the microenvironment of infarcted myocardium contributing to angiogenesis and anti-inflammation. Cell Physiol Biochem.

[16] Chen Y, Zhao Y, Chen W (2017). MicroRNA-133 overexpression promotes the therapeutic efficacy of mesenchymal stem cells on acute myocardial infarction. Stem Cell Res Ther.

[17] Chen W, Huang Z, Jiang X, et al. Overexpression of myeloid differentiation protein 88 in mice induces mild cardiac dysfunction, but no deficit in heart morphology. Braz J Med Biol Res. 2016;49:e4794. 10.1590/1414-431X20154794 PMC4681416 26628395

[18] Xiao Y, Zhang Y, Chen Y, et al. Inhibition of microRNA-9-5p protects against cardiac remodeling following myocardial infarction in mice. Hum Gene Ther. 2018. 10.1089/hum.2018.059. 10.1089/hum.2018.059 30101604

[19] Zhou Y, Jiang X, Gu P (2012). Gsdma3 mutation causes bulge stem cell depletion and alopecia mediated by skin inflammation. Am J Pathol.

[20] Wu J, Wang J, Zeng X (2016). Protein phosphatase 2A regulatory subunit B56β modulates erythroid differentiation. Biochem Biophys Res Commun.

[21] Huang Z, Ruan H-B, Xian L (2014). The stem cell factor/Kit signalling pathway regulates mitochondrial function and energy expenditure. Nat Commun.

[22] Li J, Liu S, Cao G (2018). Nicotine induces endothelial dysfunction and promotes atherosclerosis via GTPCH1. J Cell Mol Med.

[23] Dominici M, Le Blanc K, Mueller I (2006). Minimal criteria for defining multipotent mesenchymal stromal cells. The International Society for Cellular Therapy position statement. Cytotherapy.

[24] Miao C, Lei M, Hu W (2017). A brief review: the therapeutic potential of bone marrow mesenchymal stem cells in myocardial infarction. Stem Cell Res Ther.

[25] Talman V, Ruskoaho H (2016). Cardiac fibrosis in myocardial infarction—from repair and remodeling to regeneration. Cell Tissue Res.

[26] Prabhu SD, Frangogiannis NG (2016). The biological basis for cardiac repair after myocardial infarction: from inflammation to fibrosis. Circ Res.

[27] Nahrendorf M, Swirski FK, Aikawa E (2007). The healing myocardium sequentially mobilizes two monocyte subsets with divergent and complementary functions. J Exp Med.

[28] Sutton MG, Sharpe N (2000). Left ventricular remodeling after myocardial infarction: pathophysiology and therapy. Circulation.

[29] Nahrendorf M, Pittet MJ, Swirski FK (2010). Monocytes: protagonists of infarct inflammation and repair after myocardial infarction. Circulation.

[30] Ismahil MA, Hamid T, Bansal SS (2014). Remodeling of the mononuclear phagocyte network underlies chronic inflammation and disease progression in heart failure: critical importance of the cardiosplenic axis. Circ Res.

[31] Roura S, Galvez-Monton C, Mirabel C (2017). Mesenchymal stem cells for cardiac repair: are the actors ready for the clinical scenario?. Stem Cell Res Ther.

[32] Tania NP, Maarsingh H, IS TB (2017). Endothelial follistatin-like-1 regulates the postnatal development of the pulmonary vasculature by modulating BMP/Smad signaling. Pulm Circ.

[33] Broughton KM, Sussman MA (2016). Empowering adult stem cells for myocardial regeneration V2.0 success in small steps. Circ Res.

[34] Wen Z, Zheng S, Zhou C (2011). Repair mechanisms of bone marrow mesenchymal stem cells in myocardial infarction. J Cell Mol Med.

[35] Wen Z, Zheng S, Zhou C (2012). Bone marrow mesenchymal stem cells for post-myocardial infarction cardiac repair: microRNAs as novel regulators. J Cell Mol Med.

[36] Mohsin S, Troupes CD, Starosta T (2015). Unique features of cortical bone stem cells associated with repair of the injured heart. Circ Res.

[37] Holmfeldt P, Ganuza M, Marathe H (2016). Functional screen identifies regulators of murine hematopoietic stem cell repopulation. J Exp Med.

[38] Guo R, Ye X, Yang J (2018). Feeders facilitate telomere maintenance and chromosomal stability of embryonic stem cells. Nat Commun.

[39] Mangi AA, Noiseux N, Kong D (2003). Mesenchymal stem cells modified with Akt prevent remodeling and restore performance of infarcted hearts. Nat Med.

[40] Wei K, Serpooshan V, Hurtado C (2015). Epicardial FSTL1 reconstitution regenerates the adult mammalian heart. Nature.

[41] Liu S, Wang L, Wang W (2006). TSC-36/FRP inhibits vascular smooth muscle cell proliferation and migration. Exp Mol Pathol.

[42] Miyabe M, Ohashi K, Shibata R (2014). Muscle-derived follistatin-like 1 functions to reduce neointimal formation after vascular injury. Cardiovasc Res.

[43] Chan QK, Ngan HY, Ip PP (2009). Tumor suppressor effect of follistatin-like 1 in ovarian and endometrial carcinogenesis: a differential expression and functional analysis. Carcinogenesis.

