# Correction: Follistatin-like 1 protects mesenchymal stem cells from hypoxic damage and enhances their therapeutic efficacy in a mouse myocardial infarction model

**DOI:** 10.1186/s13287-024-03662-y

**Published:** 2024-02-20

**Authors:** Han Shen, Guanghao Cui, Yanqiong Li, Wenxue Ye, Yimin Sun, Zihan Zhang, Jingjing Li, Guiying Xu, Xiansheng Zeng, Yanxia Zhang, Wencheng Zhang, Zan Huang, Weiqian Chen, Zhenya Shen

**Affiliations:** 1 Department of Cardiovascular Surgery of the First Affiliated Hospital, Institute for Cardiovascular Science, Soochow University, Suzhou, 215006 China; 2 Department of Cardiology of the First Affiliated Hospital, Soochow University, Suzhou, 215006 China; 3 The Key Laboratory of Cardiovascular Remodeling and Function Research, Chinese Ministry of Education and Chinese Ministry of Health, Qilu Hospital of Shandong University, Jinan, China; 4 Jiangsu Province Key Laboratory of Gastrointestinal Nutrition and Animal Health, College of Animal Science and Technology, Nanjing Agriculture University, Nanjing, 210000 China

**Correction : Stem Cell Research & Therapy (2019) 10:17** 10.1186/s13287-018-1111-y

Following publication of this article [1], the authors found that incorrect representative echocardiographic images were mistakenly included in Fig. 5a (MSCs-mCherry group and MSCs-Fstl1 group) during manuscript preparation, which partially overlapped with representative echocardiographic images in our other contemporaneous article (Fig. 4A, MI/antagomir NC group, MI/miR-9-5p antagomir group) [2]. In response, the authors updated all representative images in Fig. 5a and re-evaluated all raw images, generating accompanying Fig. 5b–g with dot-plots indicated. All raw echocardiographic images related to Fig. 5a–g were provided as supplementary (Additional file 1: Figure S1). Notably, the main conclusions of this article remain consistent throughout.

### Supplementary Information

**Additional file 1**. **Figure S1**. All raw echocardiographic images related to Figure 5a-g.
