# Supplementary material for: Follistatin-like 1 protects mesenchymal stem cells from hypoxic damage and enhances their therapeutic efficacy in a mouse myocardial infarction model
Source: Stem Cell Res Ther. 2019 Jan 11;10:17. doi: 10.1186/s13287-018-1111-y (PMC6330478; doi:10.1186/s13287-018-1111-y)
Supplement: Supplementary file 1 — Figure S1. Murine heart tissue around the injection site was collected 1 day after myocardial delivery of indicated MSCs and expression of Fstl1 was determined by qRT-PCR (n = 4). ns not significant. (PDF 115 kb) [file 13287_2018_1111_MOESM1_ESM.pdf]

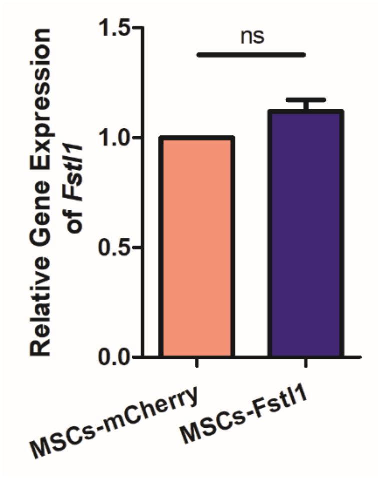

Additional file 1: Fig. S1. Murine heart tissue at the injection site was collected 1 d after myocardial delivery of indicated MSCs and expression of Fstl1 was determined by qRT-PCR (*n* = 4). ns not significant (TIF 61.8 kb).
