# Supplementary material for: Follistatin-like 1 protects mesenchymal stem cells from hypoxic damage and enhances their therapeutic efficacy in a mouse myocardial infarction model
Source: Stem Cell Res Ther. 2019 Jan 11;10:17. doi: 10.1186/s13287-018-1111-y (PMC6330478; doi:10.1186/s13287-018-1111-y)
Supplement: Supplementary file 2 — Figure S2. Echocardiographic parameters regarding wall thickness and LV mass of post-MI hearts. IVS;d (a), IVS;s (b), LVPW;d (c), LVPW;s (d), and LV mass (e) were analyzed on post-therapy 7 days respectively (n = 7–10). **P < 0.01. IVS;d interventricular septum in diastole, IVS;s interventricular septum in systole, LVPW;d left ventricular posterior wall in diastole, LVPW;s left ventricular posterior wall in systole, LV mass left ventricular mass, ns not significant. (PDF 197 kb) [file 13287_2018_1111_MOESM2_ESM.pdf]

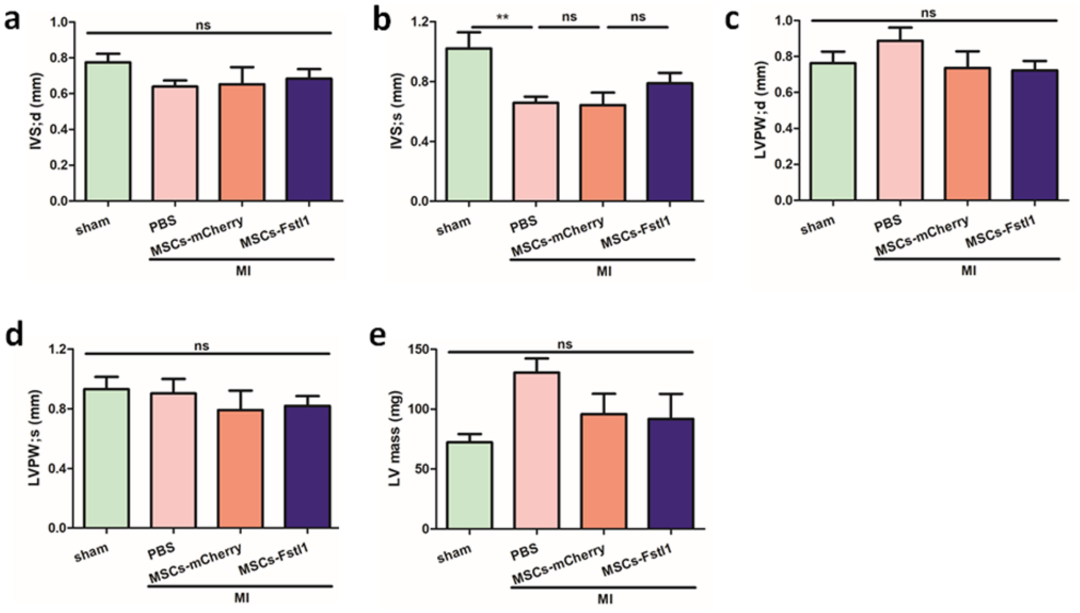

Additional file 2: Fig. S2. Echocardiographic parameters regarding wall thickness and LV mass of post-MI hearts. IVS;d (**a**), IVS;s (**b**), LVPW;d (**c**), LVPW;s (**d**), and LV mass (**e**) were analyzed on post-therapy 7d respectively (*n* = 7 - 10). ***P* < 0.01. IVS;d interventricular septum in diastole, IVS;s interventricular septum in systole, LVPW;d left ventricular posterior wall in diastole, LVPW;s left ventricular posterior wall in systole, LV mass left ventricular mass, ns not significant (TIF 208 kb).
