# Supplementary material for: Follistatin-like 1 protects mesenchymal stem cells from hypoxic damage and enhances their therapeutic efficacy in a mouse myocardial infarction model
Source: Stem Cell Res Ther. 2019 Jan 11;10:17. doi: 10.1186/s13287-018-1111-y (PMC6330478; doi:10.1186/s13287-018-1111-y)
Supplement: Supplementary file 3 — Figure S3. Changing tendency of post-MI EF (a) and FS (b) on indicated time points were determined (n = 6–7). *MI/MSCs-Fstl1 vs MI/MSCs-mCherry, P < 0.05; **MI/MSCs-Fstl1 vs MI/MSCs-mCherry, P < 0.01; &&&MI/PBS vs sham, P < 0.001. EF ejection fraction, FS fractional shortening. (PDF 164 kb) [file 13287_2018_1111_MOESM3_ESM.pdf]

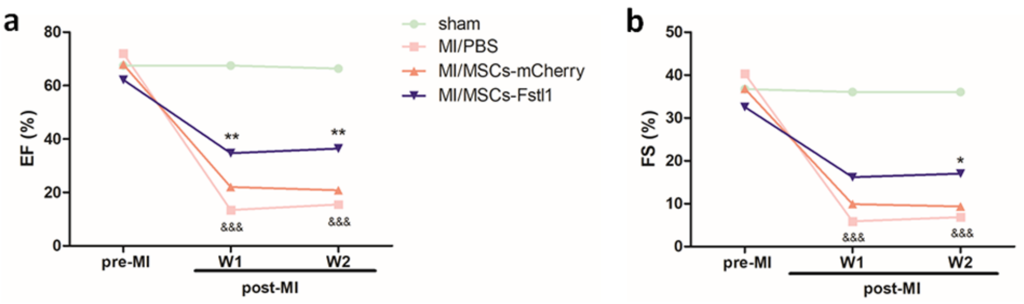

Additional file 3: Fig. S3. Changing tendency of post-MI EF (**a**) and FS (**b**) on indicated timepoints were determined (*n* = 6 - 7) *MI/MSCs-Fstl1 vs MI/MSCs-mCherry, *P* < 0.05; **MI/MSCs-Fstl1 vs MI/MSCs-mCherry, *P* < 0.01; $^{\&\&\&}$MI/PBS vs sham, *P* < 0.001 (TIF 111 kb).
