# Supplementary material for: Follistatin-like 1 protects mesenchymal stem cells from hypoxic damage and enhances their therapeutic efficacy in a mouse myocardial infarction model
Source: Stem Cell Res Ther. 2019 Jan 11;10:17. doi: 10.1186/s13287-018-1111-y (PMC6330478; doi:10.1186/s13287-018-1111-y)
Supplement: Supplementary file 4 — Figure S4. qRT-PCR analysis of CTGF in peri-infarct myocardium on post-therapy 7 days (n = 4). ***P < 0.001. CTGF connective tissue growth factor, ns not significant. (PDF 115 kb) [file 13287_2018_1111_MOESM4_ESM.pdf]

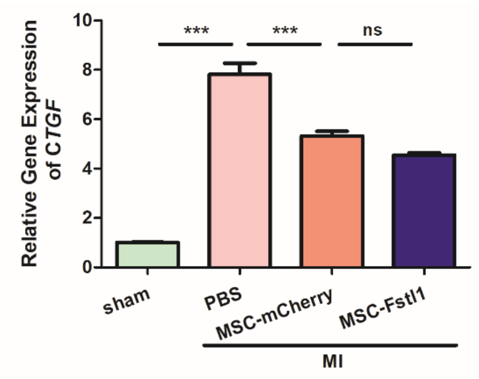

Additional file 4: Fig. S4. qRT-PCR analysis of *CTGF* in peri-infarct myocardium on post-therapy 7d (*n* = 4). ***$P < 0.001$. *CTGF* connective tissue growth factor, ns not significant. (TIF 78.3 kb)
