# Supplementary material for: Follistatin-like 1 protects mesenchymal stem cells from hypoxic damage and enhances their therapeutic efficacy in a mouse myocardial infarction model
Source: Stem Cell Res Ther. 2019 Jan 11;10:17. doi: 10.1186/s13287-018-1111-y (PMC6330478; doi:10.1186/s13287-018-1111-y)
Supplement: Supplementary file 5 — Figure S5. Serum TNF-α (a) and IL-1β (b) on post-therapy 7 days was determined by ELISA (n = 4). TNF-α tumor necrosis factor-α, IL-1β interleukin-1β, ns not significant. (PDF 131 kb) [file 13287_2018_1111_MOESM5_ESM.pdf]

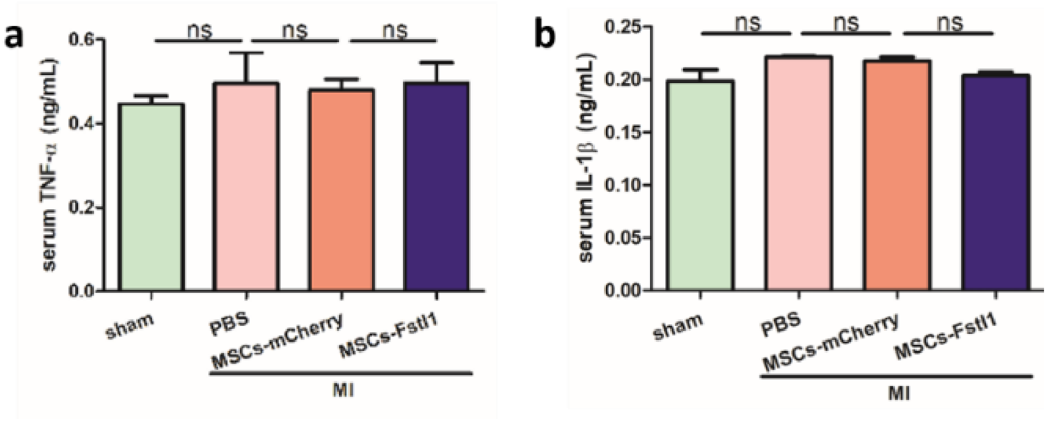

Additional file 5: Fig. S5. Serum TNF-α (a) and IL-1β (b) on post-therapy 7d was determined by ELISA (*n* = 4). TNF-α tumor necrosis factor-α, IL-1β interleukin-1β, ns not significant (TIF 182 kb).
