# Supplementary material for: Follistatin-like 1 protects mesenchymal stem cells from hypoxic damage and enhances their therapeutic efficacy in a mouse myocardial infarction model
Source: Stem Cell Res Ther. 2019 Jan 11;10:17. doi: 10.1186/s13287-018-1111-y (PMC6330478; doi:10.1186/s13287-018-1111-y)
Supplement: Supplementary file 6 — Figure S6. qRT-PCR analysis of M1 (iNOS, CD80) and M2 markers (Argnase-1, CD206) in peri-infarct myocardium on post-therapy 7 days (n = 3–4). (PDF 128 kb) [file 13287_2018_1111_MOESM6_ESM.pdf]

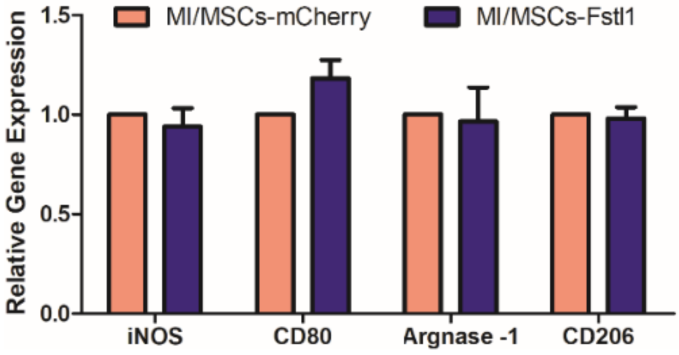

Additional file 6: Fig. S6. qRT-PCR analysis of M1 (*iNOS*, *CD80*) and M2 markers (*Argnase-1*, *CD206*) in peri-infarct myocardium on post-therapy 7d (*n* = 3 - 4). (TIF 92.2 kb)
