# Supplementary material for: Follistatin-like 1 protects mesenchymal stem cells from hypoxic damage and enhances their therapeutic efficacy in a mouse myocardial infarction model
Source: Stem Cell Res Ther. 2019 Jan 11;10:17. doi: 10.1186/s13287-018-1111-y (PMC6330478; doi:10.1186/s13287-018-1111-y)
Supplement: Supplementary file 7 — Figure S7. Representative images (a) and quantification (b) of CD31 staining within the infarct border zone on post-therapy 7 days. Scale bar = 50 μm. (n = 4–5). (PDF 142 kb) [file 13287_2018_1111_MOESM7_ESM.pdf]

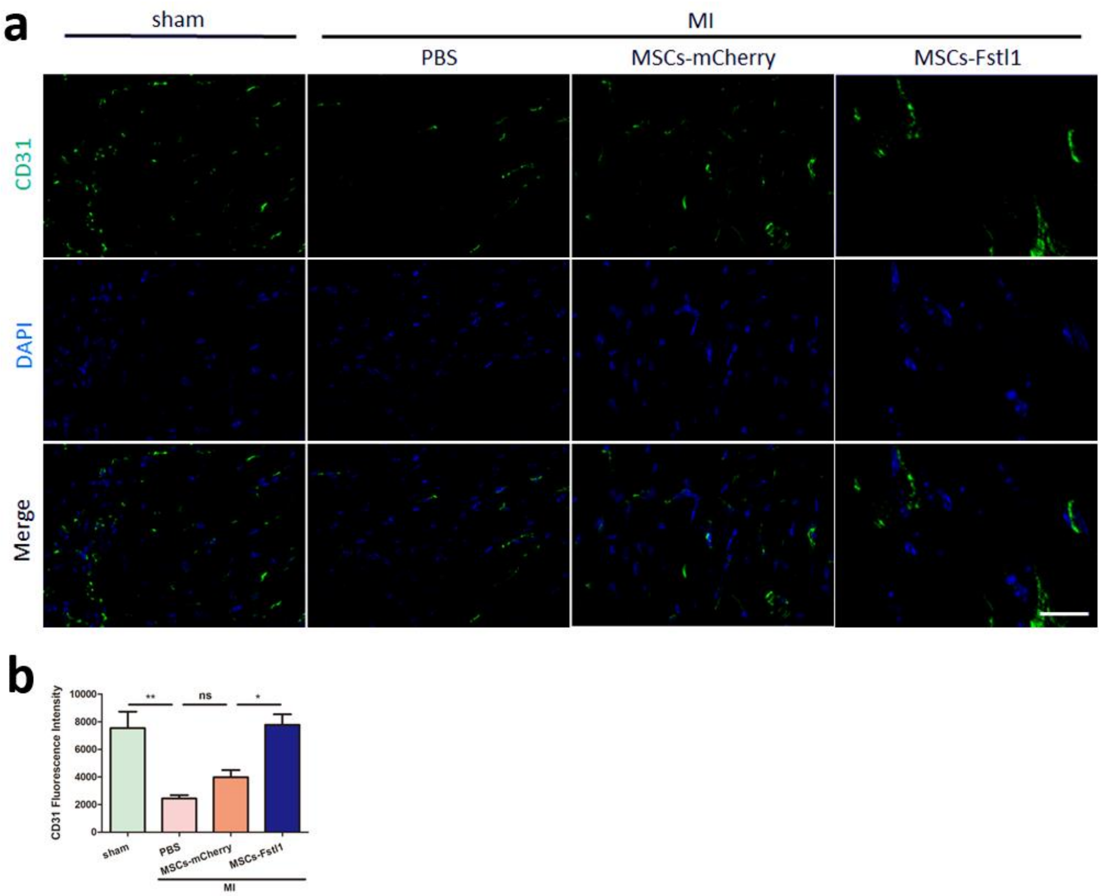

Additional file 7: Fig. S7. Representative images (a) and quantification (b) of CD31 staining within the infarct border zone on post-therapy 7d. Scale bar = 50 µm. (*n* = 4 – 5). (TIF 182 kb)
