# Supplementary material for: Follistatin-like 1 protects mesenchymal stem cells from hypoxic damage and enhances their therapeutic efficacy in a mouse myocardial infarction model
Source: Stem Cell Res Ther. 2019 Jan 11;10:17. doi: 10.1186/s13287-018-1111-y (PMC6330478; doi:10.1186/s13287-018-1111-y)
Supplement: Supplementary file 8 — Figure S8. qRT-PCR analysis of VEGF, PDGF-BB, IGF-1, Ang-1, and bFGF in hypoxic MSCs (n = 3–4). **P < 0.01. VEGF vascular endothelial growth factor, PDGF-BB platelet-derived growth factor-BB, IGF-1 insulin-like growth factor 1, Ang-1 angiopoietin-1, bFGF fibroblast growth factor-basic, ns not significant. (PDF 133 kb) [file 13287_2018_1111_MOESM8_ESM.pdf]

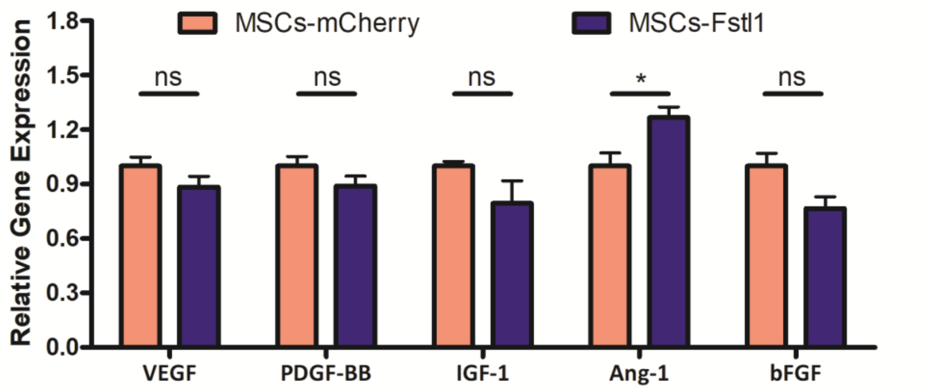

Additional file 8: Fig. S8. qRT-PCR analysis of *VEGF*, *PDGF-BB*, *IGF-1*, *Ang-1*, and *bFGF* in hypoxic MSCs ($n = 3 - 4$). **$P < 0.01$. *VEGF* vascular endothelial growth factor, *PDGF-BB* platelet derived growth factor-BB, *IGF-1* Insulin-like growth factor 1, *Ang-1* angiopoietin-1, *bFGF* basic fibroblast growth factor, ns not significant. (TIF 97.7 kb)
