# Supplementary figures and images for: Correction: Follistatin-like 1 protects mesenchymal stem cells from hypoxic damage and enhances their therapeutic efficacy in a mouse myocardial infarction model
Source: Stem Cell Res Ther. 2024 Feb 20;15:47. doi: 10.1186/s13287-024-03662-y (PMC10877755; doi:10.1186/s13287-024-03662-y)

Additional file 1: Figure S1. All raw echocardiographic images related to Figure 5a-g


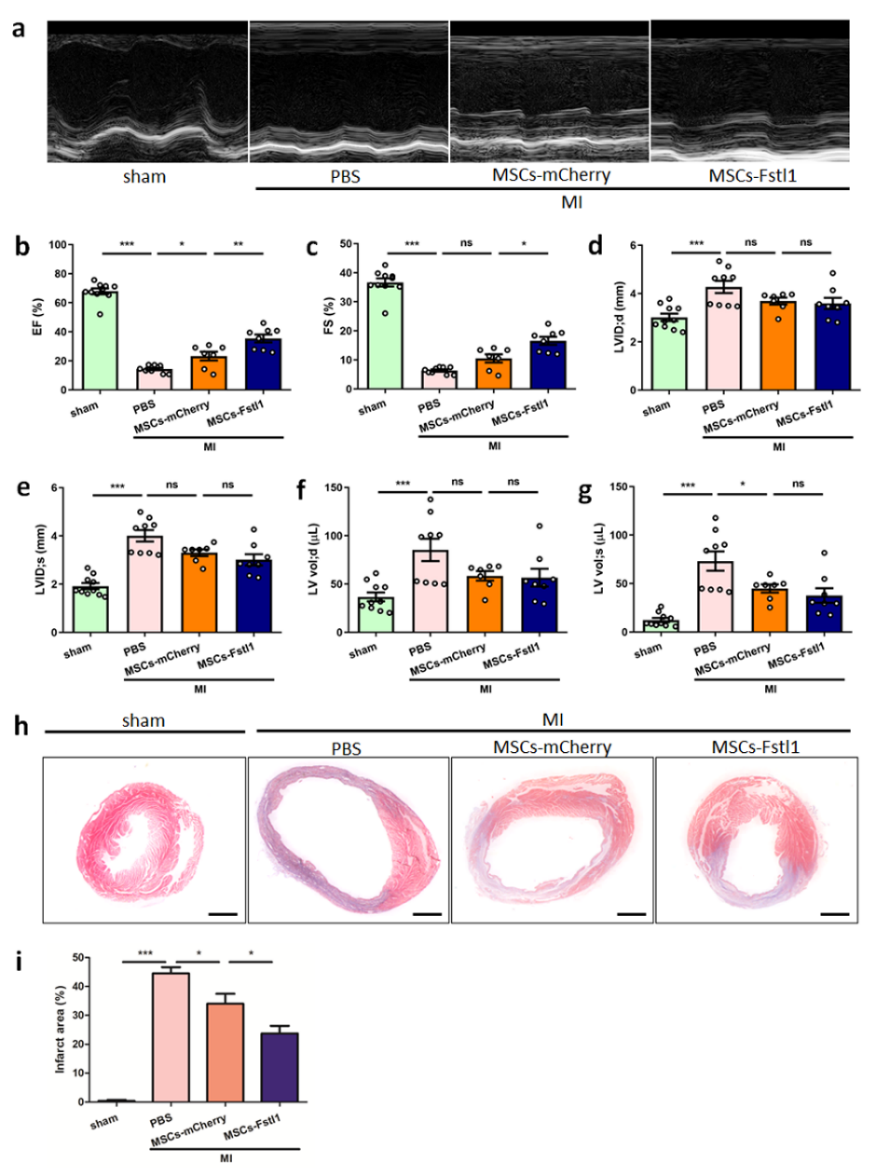

Supplement: Supplementary file 1 — Additional file 1. Figure S1. All raw echocardiographic images related to Figure 5a-g. [file 13287_2024_3662_MOESM1_ESM.docx]
